# Development of rice bran-derived nanoparticles with excellent anti-cancer activity and their application for peritoneal dissemination

**DOI:** 10.1186/s12951-024-02381-z

**Published:** 2024-03-16

**Authors:** Daisuke Sasaki, Hinako Suzuki, Kosuke Kusamori, Shoko Itakura, Hiroaki Todo, Makiya Nishikawa

**Affiliations:** 1 Laboratory of Biopharmaceutics, Faculty of Pharmaceutical Sciences, Tokyo University of Science, Yamazaki, Noda, Chiba 2641, 278-8510 Japan; 2 Laboratory of Cellular Drug Discovery and Development, Faculty of Pharmaceutical Sciences, Tokyo University of Science, Yamazaki, Noda, Chiba 2641, 278-8510 Japan; 3 Faculty of Pharmacy and Pharmaceutical Sciences, Josai University, 1-1 Keyakidai, Sakado, Saitama 350-0295 Japan

**Keywords:** Rice bran, Cancer therapy, Plant-derived nanoparticles, Apoptosis, Drug delivery system

## Abstract

**Background:**

Rice bran a by-product of the rice milling process is currently underutilized. Recent studies have shown that plant-derived nanoparticles (pdNPs) can be mass-produced at a low cost and exhibit biological and therapeutic activities. Rice bran contains various anti-cancer compounds, including γ-oryzanol and γ-tocotrienol, and rice bran-derived nanoparticles (rbNPs) can be employed as novel therapeutic agents for cancer treatment.

**Results:**

Koshihikari rice bran was suspended in water, and the suspension was centrifuged and filtered through a 0.45-µm-pore size syringe filter. The filtrate was ultracentrifuged, and the precipitates were suspended to obtain rbNPs. The rbNPs were negatively charged exosome-like nanoparticles with an average diameter of approximately 130 nm. The rbNPs exhibited cytotoxic activities against cancer cells but not against normal cells. The cytotoxic activity of rbNPs to murine colon adenocarcinoma colon26 cells was significantly greater than DOXIL^®^ or other pdNPs. The rbNPs induced cell cycle arrest and apoptosis, and reduced the expression of proliferative proteins, including β-catenin and cyclin D1. Intraperitoneal injections of rbNPs into mice bearing peritoneal dissemination of colon26 cells significantly suppressed tumor growth with no significant adverse effects.

**Conclusion:**

These results indicated that rbNPs are promising nanoparticles, hold significant potential for anti-cancer applications, and are expected to play a vital role in cancer treatment.

**Supplementary Information:**

The online version contains supplementary material available at 10.1186/s12951-024-02381-z.

## Background

Nanotechnology has various medical applications such as diagnosis [1–3], therapy [4, 5], and theranostics [6, 7], and nanoparticle-based therapy is currently advancing. In recent years, extracellular vesicles (EVs) have attracted significant attention [8–10]. EVs are nanoparticles released by cells and are composed of proteins, lipids, and nucleic acids [11, 12]. They are shuttled from extracellular vesicular-tubular structures or multivesicular bodies to the extracellular space, such as the apoplast and suberin lamellae [13, 14]. Released EVs play a prominent role in cell-to-cell communication or plant-microbe interactions by transporting functional molecules [15–17]. Isolated EVs from various parts of plants, including roots, leaves, fruits, flowers, and bran, are referred to as plant-derived nanoparticles (pdNPs) and have been extensively investigated in recent years for their development as therapeutic agents because of their unique biological and physiological activities [18–21]. For example, some pdNPs exhibit therapeutic effects on inflammatory bowel disease and colitis [22–25], while others suppress the proliferation of cancer cells and/or activated immune cells [26–31].

Several attempts have been made to use pdNPs as natural therapeutic NPs or nanoparticulate drug delivery systems for disease treatment. Surface modifications have been applied to some pdNPs to control their surface properties [32–34]. Therapeutic agents such as oligonucleotide therapeutics have also been encapsulated into pdNPs [35–37]. Recently, some clinical trials using pdNPs have been conducted [NCT01294072, NCT03493984, NCT04879810, and NCT01668849]. However, to date, no pdNPs have been approved as therapeutic agents owing to their insufficient pharmacological activity. Therefore, screening pdNPs with potent pharmacological activities is required.

Rice bran is produced in large quantities as a byproduct of rice production, most of which is not utilized and discarded [38]. However, rice bran contains nutrients, including essential fatty acids, proteins, minerals, and vitamins [39, 40]. Furthermore, rice bran has been reported to contain several anti-cancer compounds, such as γ-oryzanol, γ-tocotrienol, and tricin [41, 42]. Consequently, we hypothesized that rice bran-derived NPs or rbNPs contain anti-cancer compounds and possess substantial therapeutic potential for cancer treatment.

Therefore, in the present study, rbNPs were developed from Koshihikari rice bran, and their physicochemical and biological properties were examined. Since rbNPs efficiently inhibited the proliferation of cancer cells, their effects on the peritoneal dissemination of colon26 colon adenocarcinoma cells were examined in tumor-bearing mice. The present study demonstrates the high-yield extraction of rbNPs from rice bran and their effectiveness in inhibiting cancer cell proliferation both in vitro and in vivo.

## Methods

### Materials

Fetal bovine serum (FBS) was obtained from Biosera (East Sussex, UK). Sodium phosphotungstate and 4% paraformaldehyde phosphate buffer solution (PFA), and 30% (w/v)-acrylamide/bis mixed solutions were obtained from Nacalai Tesque, Inc. (Kyoto, Japan). Phosphatidylcholine (PC), cholesterol, and phosphatidylserine (PS) were purchased from Nippon Fine Chemical Co. Ltd. (Tokyo, Japan). Dulbecco’s modified Eagle’s medium (DMEM) and Roswell Park Memorial Institute (RPMI) 1640 medium were purchased from Nissui Pharmaceutical Co., Ltd. (Tokyo, Japan). Actinomycin D, penicillin-streptomycin-L-glutamine solution (×100) (PSG), polyoxyethylene (20) sorbitan monolaurate (Tween 20), sodium dodecyl sulfate (SDS), and skim milk powder were obtained from Wako Pure Chemical Industries, Ltd. (Osaka, Japan). All other chemicals used were of the highest commercially available grade.

### Animals

Eight-week-old female BALB/c mice were purchased from Sankyo Labo Service Co., Inc. (Tokyo, Japan) and maintained under pathogen-free conditions. The protocols for the experiments involving animals were approved by the Institutional Animal Experimentation Committee of the Tokyo University of Science (the approval number for animal experiments: Y21022). All experiments involving animals were conducted following the principles and procedures outlined in the National Institutes of Health Guide for the Care and Use of Laboratory Animals and ARRIVE guidelines.

### Cell culture

The murine macrophage-like cell line RAW264.7, murine colon adenocarcinoma cell line colon26, and firefly luciferase (fluc) stably expressing-colon26 (colon26/fluc) cells [43] were obtained from Professor Yoshinobu Takakura (Department of Biopharmaceutics and Drug Metabolism, Graduate School of Pharmaceutical Sciences, Kyoto University, Kyoto, Japan), and cultured in RPMI medium Supplemented with 10% heat-inactivated FBS and PSG at 37 °C in humidified air containing 5% CO_2_. The murine melanoma cell line B16-BL6, canine cervical cell line MDCK, human cervical adenocarcinoma cell line HeLa, and human keratinocyte cell line HaCaT were cultured in DMEM Supplemented with 10% heat-inactivated FBS and PSG at 37 °C in humidified air containing 5% CO_2_.

### Preparation of PS liposomes (PS-Lip)

Phosphatidylcholine, cholesterol, and phosphatidylserine were mixed at a molar ratio of 10:5:1 and dissolved in 2 mL of chloroform in a round-bottom flask. A lipid film was formed on the wall surface of the flask via solvent evaporation under reduced pressure using a vacuum pump in a water bath. Subsequently, 1 mL of phosphate-buffered saline (PBS) was added, and crude PS liposomes were prepared by sonication at 70 °C for 2 min using an ultrasonic cleaner (Sono Cleaner, Kaijo, Tokyo, Japan) [44]. They were extruded 3 times through a Whatman Nuclepore Track-Etched Membrane with a 100-nm pore size (Cytiva, Tokyo, Japan) at 70 °C. The aggregates were then removed by centrifugation at 10,000×*g* for 60 min to obtain PS-Lip. PS-Lip were used as control NPs.

### Preparation of rbNPs

Rice bran of Koshihikari rice (100 g) was suspended with 300 mL of PBS, and the suspension was stirred and centrifuged at 2,000×*g* for 20 min, 5,000×*g* for 30 min, and 10,000×*g* for one h at 4 °C. The supernatant was filtered through a 0.45 μm-pore size syringe filter (Minisart NML, Sartorius, Göttingen, Germany) to exclude rough residues, and the filtrate was collected as the rice bran (rb) juice. Then, 25 mL of the rb juice was ultra-centrifuged at 100,000×*g* for 120 min at 4 °C using Optima XL-K with an SW28 rotor (Beckman Coulter, Inc., Brea, CA, USA). The supernatant was collected and stored as the supernatant of the rbNPs (rb-sup). The precipitate was suspended in 1 mL of PBS and centrifuged at 10,000×*g* for one hour at 4 °C to remove the aggregates. The supernatant was collected and filtered through a 0.22 μm-pore size syringe filter (Minisart NML) to obtain rbNPs.

### Characterization of rbNPs

The particle size and zeta potential of rbNPs and PS-Lip were measured by dynamic light scattering (DLS) using an ELSZ-2000ZS instrument (Otsuka Electronics Co., Ltd., Osaka, Japan). The particle number and size distribution were measured by nanotracking analysis using NanoSight (Malvern Panalytical, NS300, Malvern, UK). For transmission electron microscopy (TEM) imaging, a drop of rbNPs was deposited onto the surface of a carbon-coated copper grid and negatively stained with 1% sodium phosphotungstate for one min, and the sample was dried at room temperature (approximately 20 °C). The samples were then observed using an H-7650 TEM (Hitachi High-Tech Co., Ltd., Tokyo, Japan) operated at 100 kV. The protein concentration of rbNPs was measured using a BCA Protein Assay Kit (Thermo Fisher Scientific Inc., Waltham, MA, USA) as previously reported [24–28].

### Preparation of pdNPs from ginger, grapes, and lemons

To prepare the ginger NPs, 50 g of ginger was mixed with 50 mL of PBS, crushed in a food processor until it became a paste (approximately 2 min), and filtered through gauze. For grape NPs, 10 grapes were processed in a food processor and filtered through gauze. For lemon NPs, three lemons were squeezed to extract the juice. The filtrates of the ginger paste and juice from either grapes or lemons were centrifuged at 10,000×*g* for 1 h. The supernatant fraction was filtered using a 0.45 μm-pore size syringe filter. Subsequently, 25 mL of the filtrate was ultracentrifuged at 100,000×*g* for 120 min at 4 °C using Optima XL-K with an SW28 rotor. The resulting precipitate was suspended in 1 mL of PBS and centrifuged at 10,000×*g* for 1 h at 4 °C to remove the aggregates. The supernatant was collected and filtered through a 0.22 μm-pore size syringe filter (Minisart NML) to obtain ginger, grape, and lemon NPs. Particle size and concentration were measured using Zetasizer and NanoSight, respectively.

### Phospholipid analysis by LC-MS/MS

To analyze the phospholipid components in rbNPs, the LC-MS/MS Method Package for Phospholipid Profiling (Shimadzu Co., Kyoto, Japan) was used according to the manufacturer’s instructions. The libraries of the phospholipid targets in the method package included PC, PE, PG, PI, PS, and SM. Briefly, rbNPs prepared at a concentration of 100 µg protein/mL were diluted 10-fold with methanol containing 0.1% formic acid for mass spectral analysis. These sample solutions (5 µL) were injected into a Kinetex C8 column (2.1 mm I.D. × 150 mm., 2.6 μm, Phenomenex, Torrance, CA, USA) at a flow rate of 0.5 mL/min. Samples were eluted using a gradient of mobile phases A (20 mM ammonium formate in water) and B (isopropanol:acetonitrile = 1:1 v/v). The concentration of the mobile phase B was programmed as 20% (0 min)–20% (1 min)–40% (2 min)–92.5% (25 min)–92.5% (26 min)–100% (35 min)–20% (38 min). The oven temperature was set at 45 °C. Data processing and lipid identification/quantification were performed using LabSolutions software (version 5.99 SP2; Shimadzu Co.). Analytical results were obtained from multiple reaction-monitoring transitions and were generally used for lipid analysis. The peak area ratio was calculated by dividing the area of the sample peak by that of the internal standard (IS) peak. As IS, 17:0–20:4 PI (Avanti Polar Lipids, Alabaster, AL, USA) was added to each sample at a final concentration of 0.38 µmol/L. Phospholipids with a peak area ≥ 5000 were analyzed.

### Cytotoxic assay of rbNPs

Colon26, B16-BL6, HeLa, HaCaT, MDCK, and RAW264.7 cells were seeded in 96-well culture plates at a density of 5 × 10^3^ cells/well and incubated for 24 h at 37 °C. The culture medium was replaced with a fresh medium containing various concentrations of rbNPs or PS-Lip. After 24 h of incubation, the cell number was measured using a Cell Counting Kit-8 (Dojindo Laboratories, Kumamoto, Japan). Separately, colon26 cells were seeded in a 96-well culture plate at a density of 5 × 10^3^ cells/well and incubated for 24 h at 37 °C. The culture medium was then replaced with fresh medium containing various concentrations of pdNPs, including grape NPs, ginger NPs, lemon NPs, and rbNPs, or DOXIL^®^. The cell numbers were measured as described earlier. The concentrations of rbNPs and rb-sup were adjusted to 1,000 µg protein/mL using a BCA Protein Assay Kit. The culture medium for colon26 cells was replaced with a fresh medium containing rbNPs or rb-sup. The cell numbers were measured as described above.

### Cytokine measurement by ELISA

RAW264.7 cells were seeded in 96-well culture plates at a density of 5 × 10^3^ cells/well and incubated for 24 h. The medium was replaced with fresh medium with or without various concentrations of rbNPs. After 24 h of incubation, the concentration of tumor necrosis factor (TNF)-α in the supernatant was measured using a Mouse TNF-α ELISA MAX Deluxe Set (BioLegend, San Diego, CA, USA).

### Uptake of DiI- or DiO-labeled rbNPs in colon26 cells

The rbNPs were labeled with the red fluorescent lipophilic dye, 1,1′dioctadecyl-3,3,3′,3′-tetramethylindocarbocyanine perchlorate (DiI; Thermo Fisher Scientific). Briefly, 0.1 mg/mL DiI solution (10 µL) was added to 1,000 µg/mL rbNPs (1 mL). The mixture was incubated for 30 min at 37°C and then ultracentrifuged at 100,000×*g* for 120 min at 4°C to obtain purified DiI-labeled rbNPs (DiI-rbNPs). For confocal microscopic observation, colon26 cells were seeded in eight-well chambered cover glass (IWAKI; AGC Techno Glass Co., Ltd., Chiba, Japan) at a density of 1 × 10^4^ cells/well and cultured overnight at 37°C. The medium was then replaced with fresh culture medium containing approximately 0.1–10 × 10^10^ DiI-rbNPs/mL. After one, three, and 12 h of incubation, the cells were fixed with 4% PFA for 30 min on ice and washed thrice with PBS. Subsequently, Vectashield Antifade Mounting Medium containing DAPI (Vector Laboratories Inc., Burlingame, CA, USA) was added. The cells were imaged using a Leica SP8 laser scanning confocal microscope (Leica, Wetzlar, Germany) and the LAS X Life Science software. For flow cytometric analysis, rbNPs were stained with green fluorescent lipophilic dye, 3,3’-dioctadecyloxacarbocyanine perchlorate (DiO; Thermo Fisher Scientific), and DiO-labeled rbNPs (DiO-rbNPs) were purified as described above. The medium was replaced with a fresh culture medium containing DiO-rbNPs. After three, six, 12, and 24 h of incubation, the cells fixed with 4% PFA were collected using a cell scraper and then filtered through a 70 μm cell strainer (Corning Incorporated, Corning, NY, USA). The cellular uptake of DiO-rbNPs was quantitatively analyzed using a BD FACSLyric flow cytometer (Becton Dickinson, San Jose, CA, USA) and FlowJo software ver8.7 (Becton Dickinson).

### Western blotting analysis

Colon26 cells were incubated with rbNPs or rb-sup, as described above. The cells were then washed thrice with PBS and lysed with RIPA lysis buffer. The total protein content of whole-cell lysates was determined using a BCA Protein Assay Kit according to the manufacturer’s protocol. Subsequently, 10 µg proteins of the cell lysate were loaded to a 4.5 and 10% SDS-PAGE and fractionated for 70 min, transferred to nitrocellulose membranes for 90 min. Subsequently, the membranes were blocked with blocking buffer (5% skim milk, 1% Tween 20 in 20 mmol/L Tris-buffered saline (TBS), pH 7.6) for 30 min. After washing with TBS with Tween-20 (TBST) three times (5 min each), the membranes were incubated with primary antibodies against β-catenin (06-734-25UG, Merck Millipore, Darmstadt, Germany), cyclin D1 (A19038, ABclonal, Woburn, MA, USA), and β-actin (010-27841, Wako Pure Chemical Industries, Ltd.) in blocking buffer at 4 °C overnight. The membrane was washed with TBST three times (5 min each), followed by incubation with horse radish peroxidase (HRP)-labeled anti-rabbit IgG or HRP-labeled anti-mouse IgG secondary antibodies (#7074 or #7076, Cell Signaling Technology, Inc. Danvers, MA, USA) in blocking buffer at room temperature for one h. Next, the membrane was washed three times with TBST again, and Immobilon^®^ Western Chemiluminescent HRP substrate (Merck Millipore, Darmstadt, Germany) was added, and the membrane was incubated at room temperature in the dark for 5 min. Protein bands were detected using the Invitrogen iBright Imaging System (Thermo Fisher Scientific).

### DNA laddering assay of colon26 cells

Colon26 cells (3 × 10^6^ cells) were treated with actinomycin D (1 µM), rbNP (1,000 µg protein/mL), or rb-sup (1,000 µg protein/mL) for 24 h. The genomic DNA of the cells was extracted using ApopLadder EX™ (Takara Bio Inc., Shiga, Japan) according to the manufacturer’s protocol. Total DNA was electrically separated on a 3% (w/v) agarose gel for 45 min and stained with ethidium bromide for 30 min at room temperature in the dark. DNA was visualized using the Invitrogen iBright Imaging System.

### Observation of chromatin condensation of colon26 cells

Colon26 cells were seeded in a 35 mm glass bottom dish (Matsunami Glass Ind., Ltd., Osaka, Japan) at a density of 1 × 10^5^ cells/dish and cultured overnight at 37 °C. The medium containing 1,000 µg/mL rb-sup or rbNPs was added to the cells and incubated for 24 h. The cells were fixed with 4% PFA for 30 min on ice and washed thrice with PBS. The cells were mounted on a glass slide using Vectashield Antifade Mounting Medium with DAPI and imaged using a Leica SP8 laser scanning confocal microscope.

### Analysis of anti-cancer compounds by ultra-high performance liquid chromatography (UHPLC)-mass spectrometry (MS) and gas chromatography (GC)-MS

The amounts of ferulic acid, γ-oryzanol, and γ-tocopherol were quantified using UHPLC with an Orbitrap Exploris 120 MS detector (Thermo Fisher). Briefly, rbNPs prepared at 100 µg protein/mL were diluted 10-fold with ethanol or methanol for MS analysis. These sample solutions (1 µL) were injected into an Xbridge C18 column (2.1 mm I.D. × 100 mm., 3 μm, Waters Corporation, Milford, MA, USA) at a flow rate of 0.2 mL/min. Samples were eluted using a gradient of mobile phases A (0.1% formic acid in water) and B (acetonitrile). The concentration of mobile phase B was programmed to be 5% (0 min), 100% (30 min), 100% (40 min), 5% (45 min), and 5% (55 min). The oven temperature was set at 40 °C. MS detection was performed by electrospray ionization (ESI). Positive and negative ESI modes were used for scanning. The following parameters were employed: capillary ionization voltage, 3.5 kV/-2.5 kV; and ion transfer tube temperature, 320 °C. The MS operated in scan mode m/z 70–700 and selected ion monitoring mode (SIM) (ferulic acid m/z 193[M-H]^−^, γ-tocotrienol m/z 409[M-H]^−^). γ-oryzanol and γ-tocopherol in rb-juice and rb-NPs were also quantified by LCMS-8045 (Shimadzu, Kyoto, Japan) using the same LC method. The MS was operated in scan mode at m/z 50-1000 and SIM (γ-oryzanol m/z 283[M-H]^−^, (+)-γ-tocopherol m/z 415[M-H]^−^).

In addition, the amounts of α-tocopherol and γ-tocotrienol in rb-juice and rbNPs were quantified by GC-MS. The sample was prepared as described above and analyzed on a GCMS-QP2020 (Shimadzu) equipped with a DB-1 column (0.25 mm I.D. × 15 m, 0.1 μm, Agilent Technologies, Santa Clara, CA, USA). The rbNPs prepared at 100 µg protein/mL were diluted 5-fold with ethanol and centrifuge at 3,000 rpm for 10 min. The supernatant was then purged and 1 mg/mL Biochanin A (Tokyo Chemical Industry Co., Ltd., Tokyo, Japan) was added, followed by dilution with methanol for MS analysis. The sample solutions (1 µL) were run with splitless injection utilizing helium as the carrier gas, and the oven program was held at 40 to 320 °C at a heating rate of 4 °C/min for 5 min. The interface and ion source temperatures were set to 280 °C. The MS was operated in scan mode m/z 150–700 and SIM (α-tocopherol m/z 431[M-H]^+^, γ-tocotrienol m/z 411 [M-H]^+^).

### Cell cycle analysis

Colon26 cells were seeded in 6-well culture plates at a density of 1 × 10^5^ cells and incubated for 24 h. Fresh medium containing rbNPs or rb-sup (1,000 µg protein/mL) was added. After incubation for 6 h, the cells were washed with PBS and collected using a cell scraper. To analyze the cell cycle, cell nuclei were stained with Cell Cycle Assay Solution Blue (Dojindo Laboratories) according to the manufacturer’s protocol. The cells were analyzed using a BD FACSLyric flow cytometer, and cell cycle analysis was performed using FlowJo software ver8.7.

### Anticancer effect of rbNPs in a peritoneal dissemination model mice

To prepare a mouse model of peritoneal dissemination, colon26/fluc cells suspended in PBS (2 × 10^5^ cells/100 µL) were injected intraperitoneally into BALB/c mice. The following day, the mice were randomly assigned to two treatment groups, and PBS (vehicle) or rbNPs (1 × 10^10^ NPs/shot) were injected with three cycles of three daily injections, with an injection-free day between cycles. At day 12, the mice were anesthetized with isoflurane, and 200 µL (3 mg) of VivoGlo™ Luciferin In Vivo Grade (Promega, Madison, WI, USA) was intraperitoneally injected. Luciferase activity was detected using In-Vivo Xtream (Bruker BioSpin, Billerica, MA, USA).

### Tissue distribution of rbNPs after intraperitoneal injection to mice

The rbNPs were labeled with the near-infrared fluorescent lipophilic dye, 1,1′-dioctadecyl-3,3,3′,3′-tetramethylindotricarbocyanine iodide (DiR; Thermo Fisher Scientific). DiR-labeled rbNPs (DiR-rbNPs) were prepared using the protocol described above. DiR-rbNPs (1 × 10^10^ NPs/mouse) or DiR (0.5 µg/mouse) were intraperitoneally injected into BALB/c mice. At 15 min and one, three, six, and 24 h after injection, the mice were euthanized with isoflurane, and the major organs and abdominal wall were harvested for ex vivo imaging. The fluorescence intensity of the organs was visualized using In-Vivo Xtream (Bruker BioSpin).

### Evaluation of adverse effects of rbNP after intraperitoneal injection to mice

The rbNPs were repeatedly injected into BALB/c mice, with three cycles of three daily injections and one injection-free day between cycles. Blood was collected from the orbital plexuses of mice using an animal lancet (Medipoint, Mineola, NY, USA) on days four, eight, and 12. The blood was then centrifuged at 2,000×*g* for 20 min, and the serum obtained was stored at −80 °C until subsequent experiments. The serum concentrations of tumor necrosis factor (TNF)-α and interleukin (IL)-6 were measured using the Mouse TNF-α ELISA MAX Deluxe Set (BioLegend, San Diego, CA, USA) and the Mouse IL-6 Uncoated ELISA kit (Thermo Fisher Scientific), respectively. In addition, the serum levels of Cre, AST, and ALT were measured using a LabAssay Creatinine kit (Wako Pure Chemical Industries, Ltd.) and a transaminase CII-test Wako kit (Wako Pure Chemical Industries, Ltd., Osaka, Japan), respectively.

### Statistical analysis

Statistical differences were evaluated using one-way analysis of variance (ANOVA), followed by Dunnett’s test for multiple comparisons or Student’s t-test for comparisons between two groups. Statistical significance was set at *p* < 0.05.

## Results

### Preparation and characterization of rbNPs

**Fig. 1:**
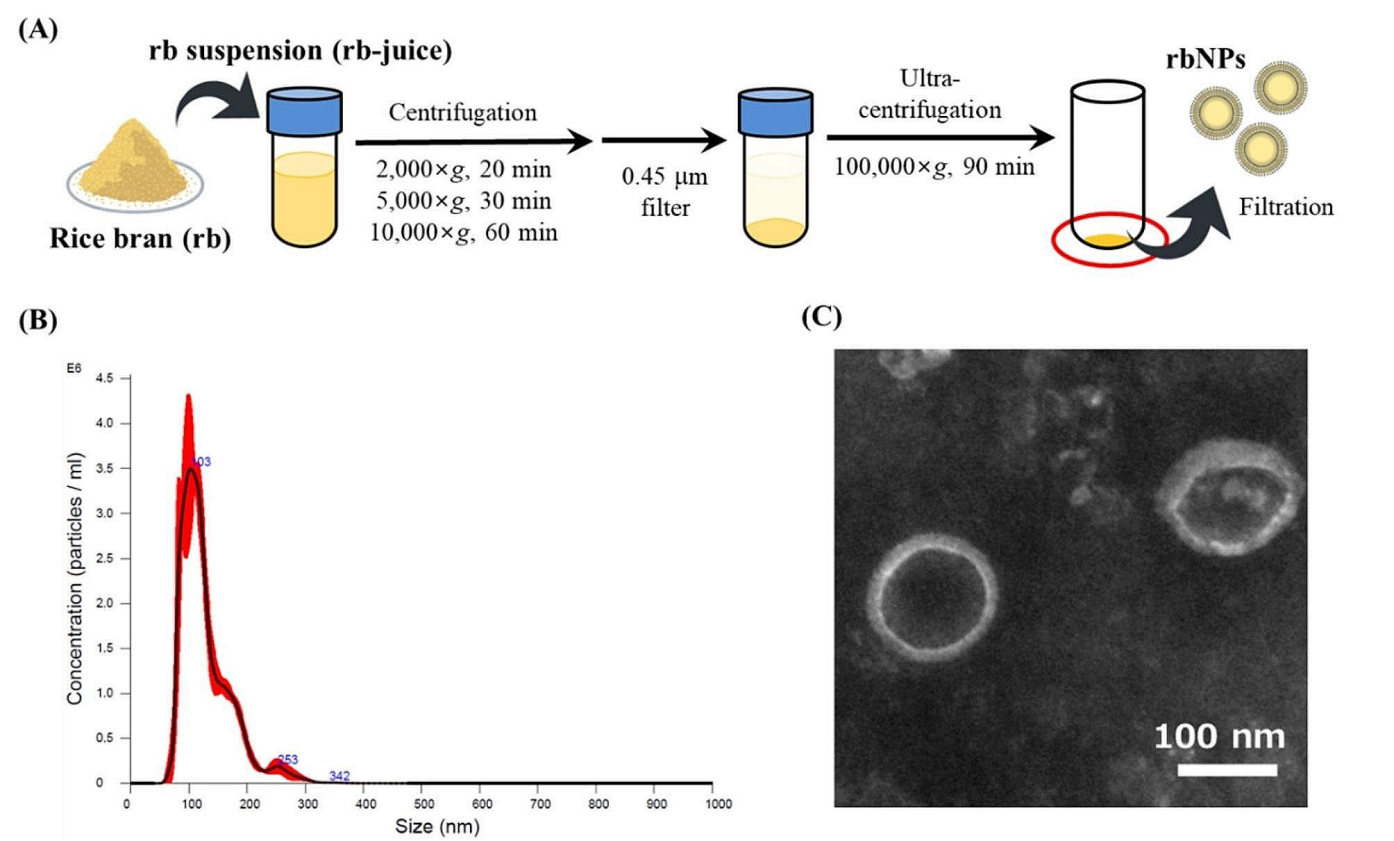
Preparation and characterization of rbNPs. **(A)** Schematic diagram of rbNP preparation. Rice bran suspension (rb-juice) was sequentially centrifuged, filtered, and ultracentrifuged to obtain rbNPs after filtration. **(B)** Size distribution of rbNPs determined by NanoSight NS300. The red area indicates the standard deviation of five measurements. **(C)** A TEM image of rbNPs. The image was obtained using H-7650 TEM. The scale bar indicates 100 nm. rbNPs, rice bran-derived nanoparticles; TEM, transmission electron microscopy

Figure 1A shows a schematic illustration of the preparation process of rbNPs from a suspension of rice bran (rb-juice) using sequential centrifugation and ultracentrifugation. Nanoparticle tracking analysis showed that the rbNPs were uniform in size, with a peak size of approximately 103 nm (Fig. 1B). TEM images revealed that the rbNPs had an EV-like hollow membrane structure (Fig. 1C). Table 1 shows the average particle size and zeta potential of the rbNPs as determined using DLS. The particle size and zeta potential of rbNPs were 139.0 ± 1.3 nm and −17.2 ± 2.2 mV, respectively. For comparison, phosphatidylserine-containing liposomes (PS-Lip) with a comparable particle size (137.4 ± 1.2 nm) and zeta potential (−15.5 ± 1.4 mV) to those of rbNPs were prepared. The average yield of rbNPs was approximately 4 × 10^13^ NPs/100 g of rice bran.

**Table 1:** Characteristics of rbNPs and PS-Lip

NPs	Particle size (nm)	Zeta potential (mV)	Particle yield (×10^13^ NPs/100 g)	
rbNPs	139.0 ± 1.3	−17.2 ± 2.2	3.9 ± 0.3	
PS-Lips	137.4 ± 1.2	−15.5 ± 1.4		

The results are expressed as the mean ± standard deviation (SD) of three independent experiments

Table 2 summarizes the number and particle size of rbNPs after storage at 4 °C. These parameters hardly changed during the four-week experimental period. Liquid chromatography coupled with tandem mass spectrometry (LC-MS/MS) analysis showed that the rbNPs contained the following phospholipids: lysophosphatidylcholines (LPCs), phosphatidylcholines (PCs), phosphatidylethanolamines (PEs), phosphatidylserine (PSs), and sphingomyelins (SMs) (Supplementary Table S1)

**Table 2:** Number and particle size of rbNPs after storage at 4°C

Day (week)	0	1	2	3	4	
Particle size (nm)	147.3 ± 6.7	155.3 ± 7.3	152.8 ± 7.4	142.0 ± 8.8	150.8 ± 19	
Particle number (× 10^10^ NPs/mL)	10.9 ± 1.1	9.9 ± 1.2	11.4 ± 2.0	13.0 ± 2.3	12.3 ± 2.7	

The results are expressed as the mean ± standard deviation (SD) of three independent experiments.

### Cytotoxic activity of rbNPs against cell lines

**Fig. 2:**
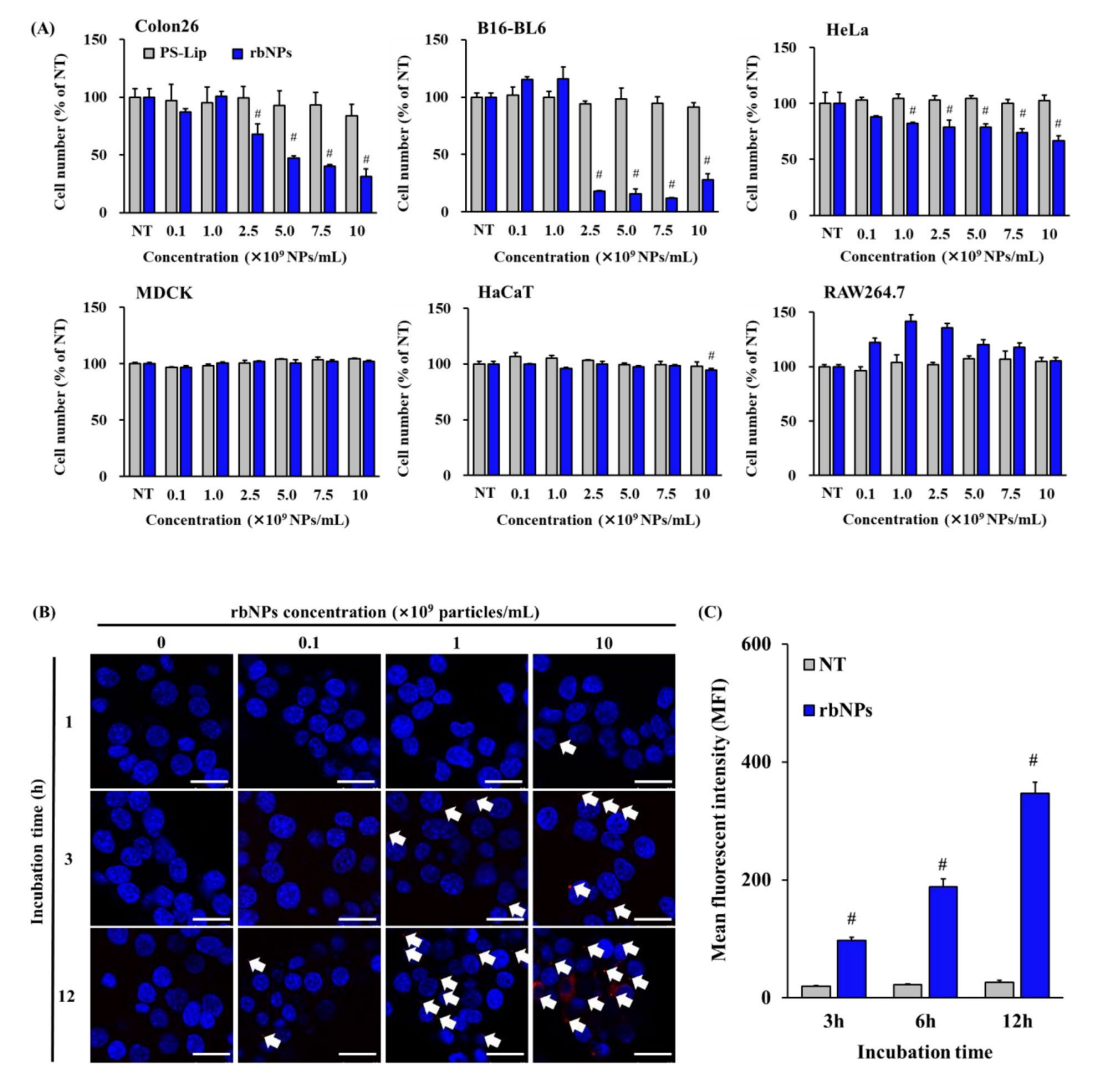
Interaction of rbNPs with culture cells. **(A)** Cell number is measured by CCK-8 assay after 24 h incubation with rbNPs or PS-Lip at varying concentrations. Colon26, B16-BL6, HeLa, MDCK, HaCaT and RAW264.7 cells are incubated with 0.1−10 × 10^9^ rbNPs or PS-Lip/mL. Results are expressed as the mean ± SD of three samples. #*p* < 0.01 vs. no treatment (NT) group. **(B)** Confocal microscopic images of colon26 cells after the addition of DiI-labeled rbNPs (DiI-rbNPs). Colon26 cells are incubated with 0.1−10 × 10^9^ DiI-rbNPs/mL for 1, 3, and 12 h. Scale bars indicate 50 μm. White arrows indicate DiI-rbNPs. **(C)** Cellular uptake of DiO-labeled rbNPs (DiO-rbNPs) in colon26 cells. Colon26 cells are incubated with DiO-rbNPs for 3, 6, 12, and 24 h at 37 °C, then fixed with paraformaldehyde. The fluorescence intensity of colon26 cells is quantified by flow cytometry, and the mean fluorescence intensity (MFI) is calculated. Results are expressed as the mean ± SD of three samples. #*p* < 0.01 vs. NT group. Colon26, murine colon adenocarcinoma cell line; B16-BL6, murine melanoma cell line; HeLa, human cervix adenocarcinoma cell line; MDCK, canine kidney cell line; HaCaT, human keratinocyte cell line; and RAW264.7, murine macrophage cell line

The cytotoxic activity of the rbNPs against cancer and non-cancerous cell lines was also examined. Figure 2A shows the cell numbers after 24 h of incubation with PS-Lip or rbNPs at varying concentrations. The addition of PS-Lip exhibited minimal effect on the number of cells, irrespective of the cell type. In contrast, rbNPs significantly reduced the number of murine colon adenocarcinoma colon26 cells, murine melanoma B16-BL6 cells, and human cervical adenocarcinoma HeLa cells in a concentration-dependent manner. The rbNPs showed no significant cytotoxicity against non-cancerous cells, including the canine kidney cell line MDCK and human keratinocyte cell line HaCaT. The number of murine macrophage-like cell line RAW264.7 cells tended to increase after adding rbNPs. Cytokine release from RAW264.7 cells was measured after the addition of rbNPs. RAW264.7 cells significantly released the proinflammatory cytokine TNF-α after incubation with rbNPs in a particle concentration-dependent manner (Supplementary Fig. S2). These results indicate that rbNPs have selective cytotoxicity towards cancer cells and stimulatory activity on macrophages.

### Uptake of rbNPs by colon26 cells

Since rbNPs significantly reduced the number of colon26 cells, the uptake of rbNPs in colon26 cells was examined using rbNPs labeled with DiI, a red fluorescent dye. Figure 2B shows the confocal images of colon26 cells after the addition of rbNPs at varying concentrations. After one hour of incubation, red fluorescence signals derived from DiI-rbNPs were hardly observed, irrespective of the rbNP concentration. As the incubation time increased to three or 12 h, the red fluorescence signals in the cells also increased, and the highest intensity was observed at 10 × 10^10^ NPs/mL at 12 h. To quantitatively evaluate the cellular uptake of rbNPs, the cells’ mean fluorescence intensity (MFI) was measured at three, six, and 12 h after incubation with DiO-rbNPs. The MFI values of the cells increased with time, indicating that rbNPs were taken up by colon26 cells in a concentration- and time-dependent manner.

### Anti-cancer activity and mechanism of rbNPs

**Fig. 3:**
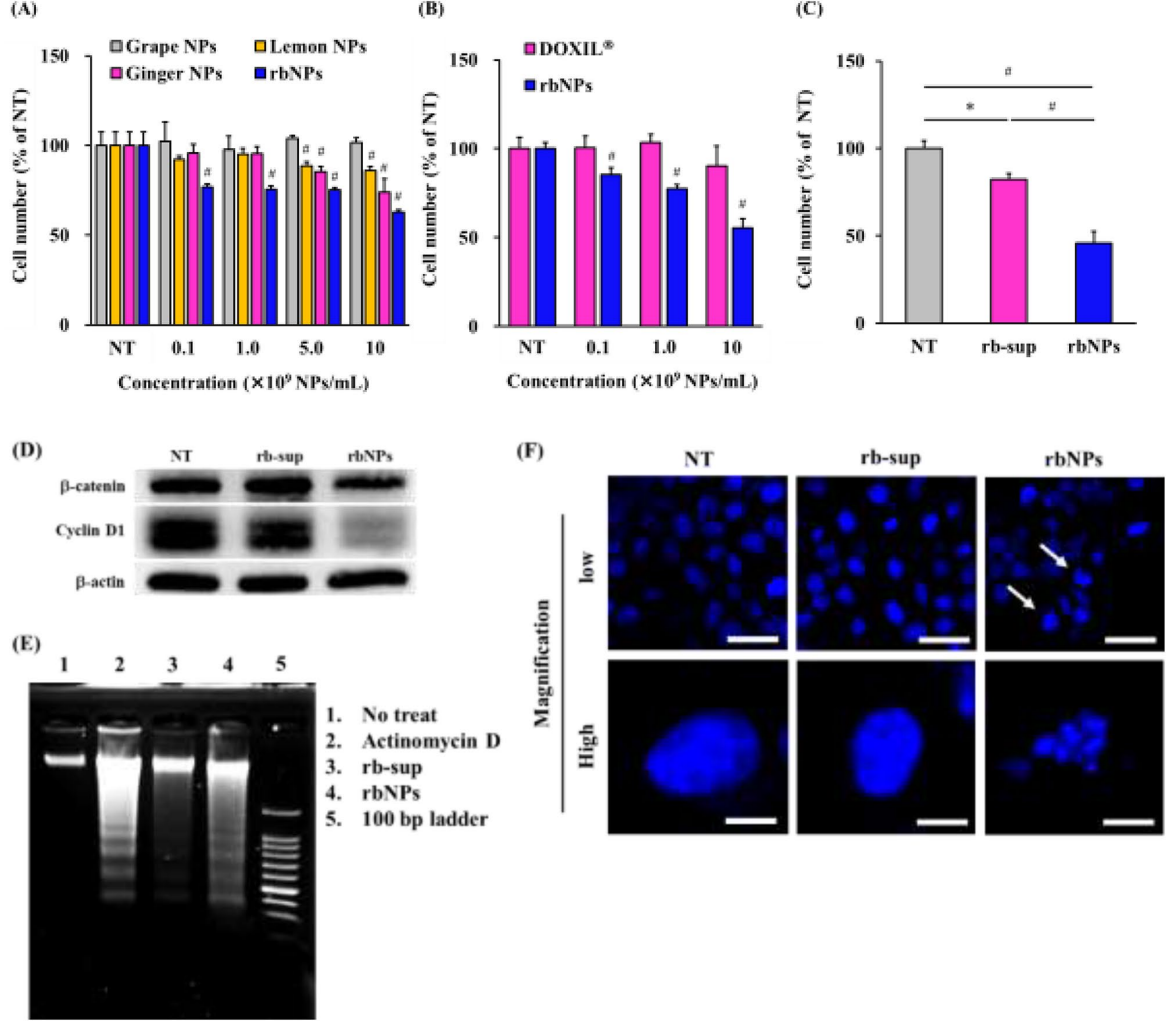
Comparison of rbNPs with other pdNPs, DOXIL^®^ or rb-sup. **(A)** The number of colon26 cells 24 h after the addition of grape, ginger, and lemon NPs, and rbNPs at varying concentrations. Colon26 cells are incubated with 0.1−10 × 10^9^ NPs/mL, and the cell number is measured at 24 h using CCK8 assay. Results are expressed as the mean ± SD of four samples. #*p* < 0.01 vs. NT group. **(B)** The number of colon26 cells 24 h after the addition of rbNPs or DOXIL^®^. Colon26 cells are incubated with 0.1−10 × 10^9^ NPs/mL of rbNP or DOXIL^®^, and the cell number is measured as described earlier. Results are expressed as the mean ± SD of three samples. #*p* < 0.01 vs. DOXIL^®^. **(C)** The number of colon26 cells 24 h after addition of rbNPs and rb-sup. Colon26 cells are incubated with 1,000 µg/mL of rbNP or rb-sup, and the cell number is measured at 24 h using CCK8 assay. Results are expressed as the mean ± SD of three samples. *p < 0.05. #*p* < 0.01. **(D)** Western blot analysis of β-catenin, cyclin D1, and β-actin in colon26 cells. Colon26 cells are treated with 1,000 µg/mL rbNP or rb-sup for 24 h, and the cellular proteins are extracted for the analysis The bands of each protein are visualized using Invitrogen iBright Imaging Systems. **(E)** DNA fragmentation of colon26 cells after addition of rbNPs, rb-sup, or actinomycin D. Colon26 cells are incubated with 1 µM actinomycin D or 1,000 µg/mL rbNP or rb-sup. The DNA of the cells is extracted and subjected to 3% agarose gel electrophoresis, followed by visualization using Invitrogen iBright Imaging Systems. **(F)** Confocal images of colon26 cells stained with DAPI. Colon26 cells are incubated with 1,000 µg/mL rb-sup or rbNP for 24 h at 37 °C, fixed with paraformaldehyde, and the nuclei of the cells are stained with DAPI. Scale bars indicate 50 μm (low magnification) and 10 μm (high magnification). White arrows indicate chromatin condensation

The cytotoxic activity of rbNPs to colon26 cells was compared with other previously reported pdNPs, DOXIL^®^, or the supernatant of rbNPs. The pdNPs from grapes, ginger, and lemon [22, 25, 45] were selected for comparison. Figure 3A shows the number of colon26 cells after 24 h of incubation with different concentrations of grape, ginger, and lemon NPs, or rbNPs, whose peak particle sizes were comparable, ranging from approximately 70 to 120 nm (Supplementary Table S3). Lemon and ginger NPs significantly reduced the number of colon26 cells at high concentrations; however, rbNPs exhibited the greatest reduction in the number of colon26 cells at all concentrations. Subsequently, the cytotoxic activity of rbNPs was compared with that of DOXIL^®^, a liposomal anti-cancer agent, on a particle number basis. Although DOXIL^®^ rarely reduced the number of colon26 cells from 0.1 to 10 × 10^9^ particles/mL, rbNPs significantly reduced the number even at a low concentration of 0.1 × 10^9^ particles/mL (Fig. 3B). Furthermore, the cytotoxic activity of rbNPs against colon26 and HaCaT cells was compared to that of doxorubicin. Doxorubicin showed cytotoxicity to both colon26 and HaCaT cells, whereas rbNPs showed cytotoxic activity only against colon26 cells (Supplementary Fig. S1A, S1B).

Next, the cytotoxic activity of the rbNPs was compared with that of the rb-sup after ultracentrifugation. Figure 3C shows the number of colon26 cells after adding rbNPs or rb-sup. The concentrations of rbNPs and rb-sup added to the cells were adjusted to the protein concentration because this concentration has often been used as an indicator of pdNPs [24–28] and the protein concentrations of these two samples were almost equivalent. The rbNPs exhibited higher cytotoxic activity against colon26 cells in comparison to rb-sup. The cytotoxic mechanism of rbNPs against colon26 cells was examined using rb-sup as a control. UHPLC-MS and GC-MS analyses showed the amounts of major anti-cancer compounds contained in rice bran, that is, ferulic acid, γ-oryzanol, α-tocopherol, γ-tocopherol, and γ-tocotrienol, in rb-juice and rbNPs. These anti-cancer compounds, except γ-tocopherol, were concentrated in the rbNPs. Figure 3D shows the expression of cellular proteins related to proliferation, cell cycle, β-catenin, and cyclin D1. The expression of these proteins was reduced by adding rbNPs but not by rb-sup. In contrast, no significant change in β-catenin expression was observed after the addition of rb-sup or rbNPs to HaCaT cells (Supplementary Fig. S1C). In addition, cell cycle analysis of colon26 cells was performed after adding rbNPs or rb-sup. Table 3 shows that adding rbNPs significantly reduced the proportions of the G1 and S phases of colon26 cells, and significantly increased the proportion of cells in the G2/M phase. Subsequently, the apoptosis of colon26 cells was examined. Actinomycin D, the positive control for apoptosis, induced DNA fragmentation (Fig. 3E). Fragmentation was also observed in rbNP-treated colon26 cells. Figure 3F shows the confocal images of colon26 cells after staining the nuclei with DAPI. High-magnification images showed that the morphology of the nuclei of the rbNP-treated colon26 cells was different from that of the no-treatment (NT) or the rb-sup-treated groups, indicating that rbNPs induced chromatin condensation.

**Table 3:** Cell cycle analysis of colon26 cells at six hours after the addition of rbNPs or rb-sup

Phase	G1	S	G2/M	
NT	43.4 ± 1.16	29.5 ± 2.08	11.0 ± 1.06	
rb-sup	41.7 ± 0.25	28.0 ± 1.13	12.3 ± 1.00	
rbNPs	36.9 ± 1.99^#^	24.9 ± 0.38*	14.7 ± 0.12^#^	

The results are expressed as the mean ± standard deviation (SD) of three independent experiments. *p < 0.05 vs. NT group. #*p* < 0.01 vs. NT group.

### Anti-cancer effect of rbNPs in peritoneal dissemination model mice

**Fig. 4:**
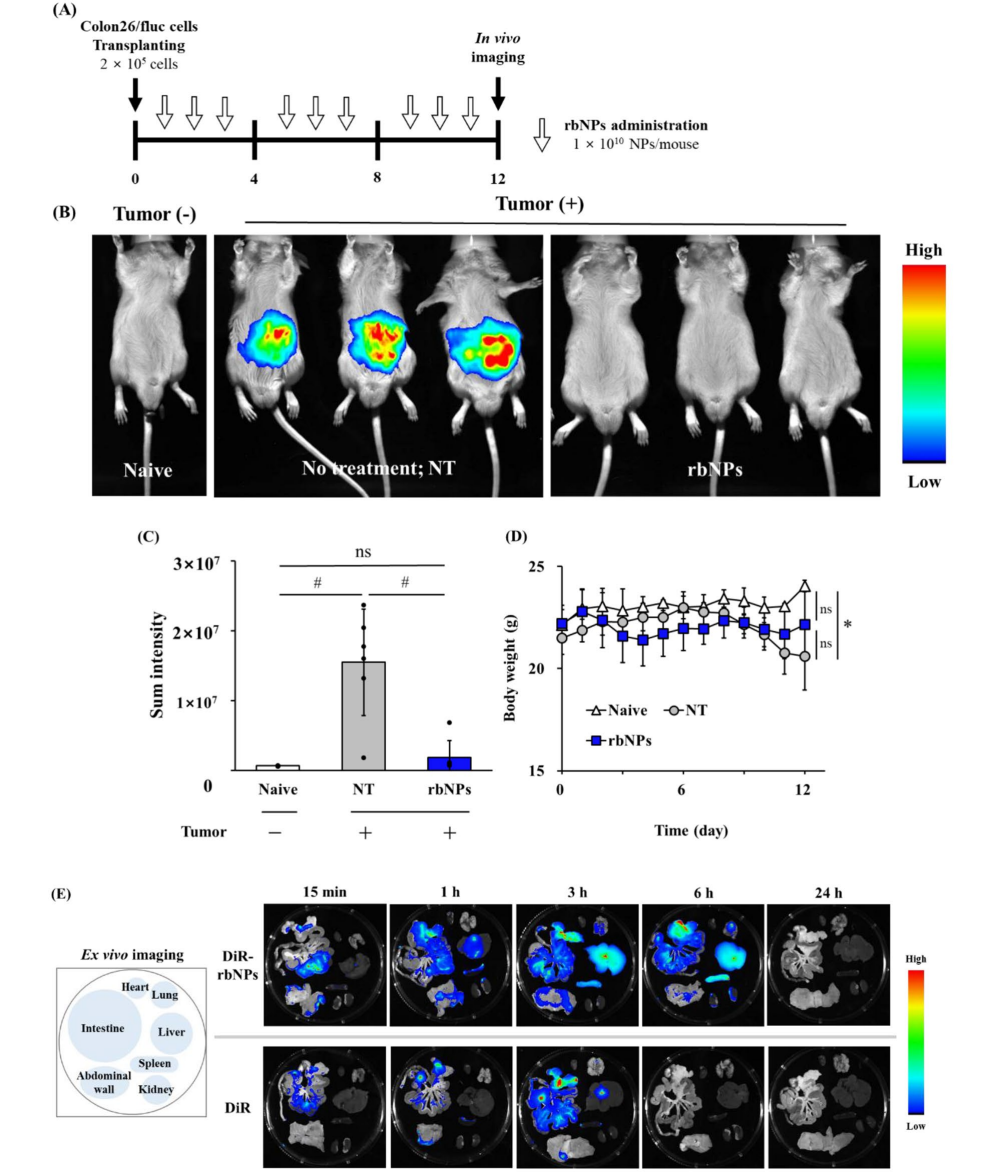
Anti-cancer effect of rbNPs in peritoneal dissemination model mice. **(A)** Flow diagram for the evaluation of the anti-cancer effect of rbNPs in peritoneal dissemination model mice. Colon26/fluc cells are transplanted to the peritoneal cavity of mice, and rbNPs are injected with three cycles of 3 daily injections and 1 injection-free day between cycles. At day 12, mice are subjected to in vivo imaging. **(B)** In vivo imaging of colon26/fluc cells in mice. Mice are anesthetized, and injected with VivoGlo™ Luciferin, and the luminescence derived from colon26/fluc cells in mice is detected. **(C)** The sum intensity of luciferase activity is calculated based on the images of Fig. 4B. Results are expressed as the mean ± SD of three or six. #*p* < 0.01. ns, not significant. **(D)** Body weight changes of mice. The body weight of mice is measured daily. Results are expressed as the mean ± SD of three or six mice. *p < 0.05 vs. NT group; ns, not significant. **(E)** Fluorescence images of mouse organs harvested 15 min, 1, 3, 6, and 24 h after injection of DiR-rbNPs or DiR. BALB/c mice are intraperitoneally injected with DiR-rbNPs or DiR. At 15 min, and one, three, six, and 24 h after injection, the fluorescence intensity of organs is visualized using an in vivo imaging system

The anti-cancer effect of the rbNPs was evaluated using a peritoneal dissemination mouse model, which was established by transplanting firefly luciferase (fluc)-expressing colon26 (colon26/fluc) cells into BALB/c mice. The rbNPs were intraperitoneally injected with three cycles of three daily injections and one injection-free day between cycles (Fig. 4A). Figure 4B shows the luminescence derived from colon26/fluc cells in mice on day 12. Strong luminescence was detected in the NT mice, whereas minimal luminescence was detected in rbNP-treated mice. The luminescence intensity of the rbNP-treated group was significantly lower than that of the NT group. No significant difference was observed between naïve (non-transplanted) and rbNP-treated mice (Fig. 4C). The body weight of mice in the NT group significantly decreased over time. In contrast, it was maintained in the rbNP-treated group (Fig. 4D). Figure 4E shows the tissue distribution of DiR-rbNPs and DiR after peritoneal injection in mice. DiR-rbNPs were detected in the intestine, liver, and spleen at least six hours post-injection. The distribution of DiR differed from that of DiR-rbNPs. These results suggest that the rbNPs can be distributed to multiple abdominal organs and retained for at least six hours.

### Adverse effects of rbNPs after repeated injections to mice

**Fig. 5:**
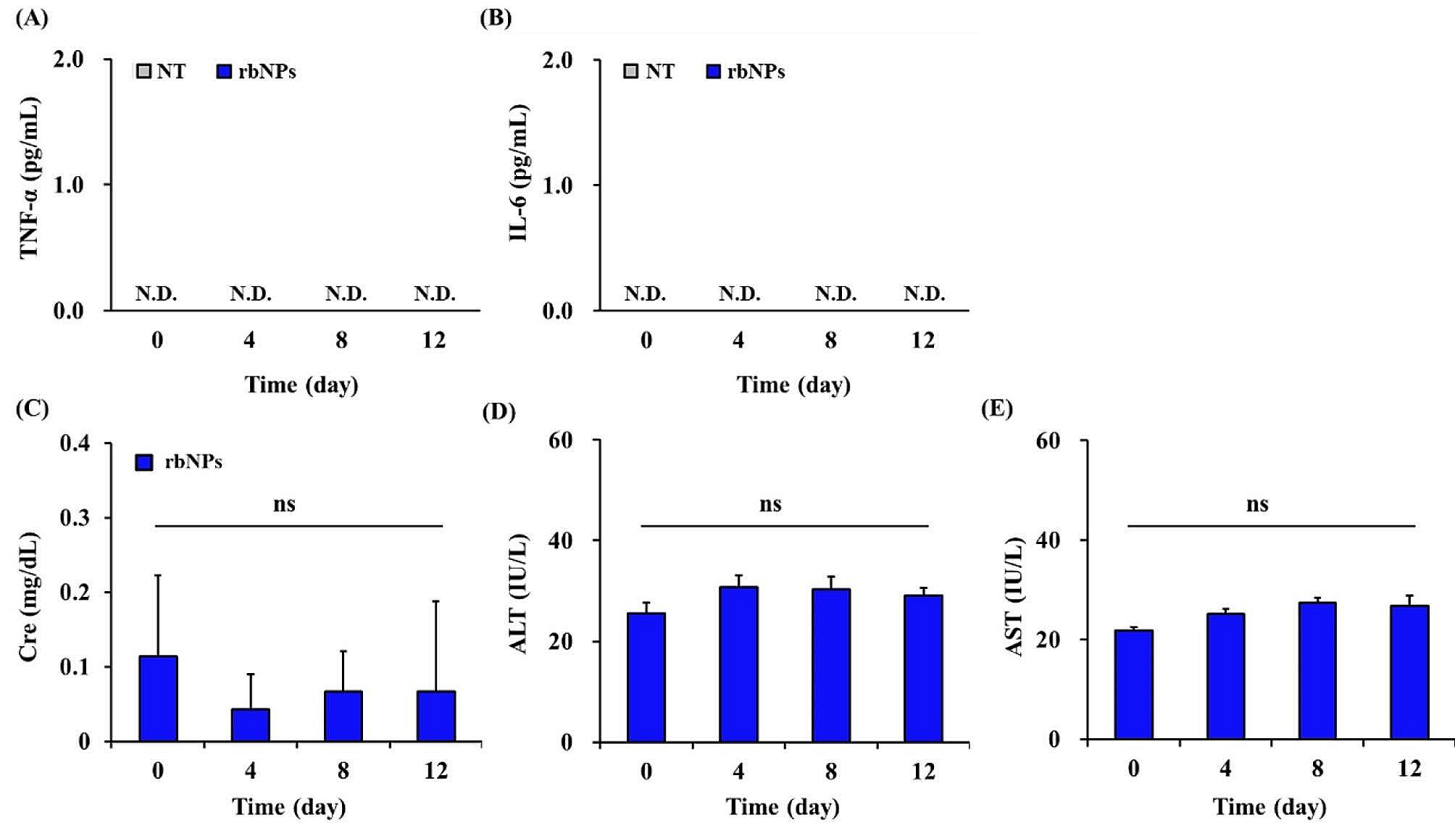
Adverse effects of rbNPs in mice. Mice are injected with rbNPs according to the same cycle described in the anti-tumor experiment. **(A-E)** The serum levels of interleukin (IL)-6, tumor necrosis factor (TNF)-α, alanine aminotransferase (ALT), aspartate aminotransferase (AST), and creatinine (Cre). Phosphate buffered saline (PBS, the vehicle) or rbNPs are injected into mice, and the blood is collected at day zero, four, eight, and 12 after the first injection. Subsequently, the serum is obtained and the levels of **(A)** TNF-α and **(B)** IL-6 are determined by ELISA. The serum levels of **(C)** Cre, **(D)** ALT, and **(E)** AST are also measured. Results are expressed as the mean ± SD of three samples. ns, not significantly different from one another; N.D., not detected. Cre, creatinine; ALT, alanine aminotransferase; AST, aspartate aminotransferase

Finally, the adverse effects of the rbNPs were examined in mice. Mice were treated using the same protocol as shown in Fig. 4A. Figure 5A and B show the serum levels of interleukin (IL) -6 and tumor necrosis factor (TNF) -α, after repeated injections of rbNPs. Neither IL-6 nor TNF-α was detected in the entire duration of 12 days of the experiment. In addition, serum creatinine (Cre), alanine aminotransferase (ALT), and aspartate aminotransferase (AST) levels did not change after the rbNP injections (Fig. 5C-E).

## Discussion

Since EV-like NPs in sunflowers were reported in 2009 [46], pdNPs have been isolated from many plants, and their biological activities have been elucidated [18–21]. TEM observations confirmed that pdNPs isolated from grape, grapefruit, ginger, and carrot had vesicular structures [47]. The rbNPs prepared from rice bran in the present study were uniform nanoparticles with a vesicular structure and an average diameter of approximately 130 nm. These physicochemical properties of the rbNPs were comparable to those of previously reported pdNPs. In addition, high-performance liquid chromatography analysis revealed that LPCs, PCs, PEs, PSs, and SMs were present in the rbNPs (Supplementary Table 1). These results and TEM images suggest that rbNPs are NPs with a lipid bilayer membrane composed of a mixture of phospholipids.

Before harnessing the therapeutic potential of pdNPs, certain concerns must be resolved [18]. One of these concerns is the preparation efficiency of pdNPs. This study obtained NPs from five different plant species, including rice bran. The number of rbNPs prepared from 100 g of rice bran was approximately 4 × 10^13^ NPs (Table 1), the highest among all the pdNPs used in this study. The preparation efficiency was also higher than that of previously reported corn-derived NPs (cNPs) [48]. This high production efficiency may be advantageous for developing therapeutic NPs. The second concern in the context of pdNPs is the low pharmacological activity in comparison to pharmaceutical drugs. However, rbNPs exhibited higher anti-cancer activity than DOXIL^®^ (Fig. 3B), a liposomal pharmaceutical formulation of doxorubicin [49, 50]. In addition, doxorubicin was cytotoxic to both colon26 cancer cells and HaCaT non-cancerous cells, whereas rbNPs were specifically cytotoxic to colon26 cells, suggesting that rbNPs are safer than doxorubicin (Supplementary Fig. S1A, S1B). Stability is an important factor in clinical applications. The present study showed that the physicochemical properties of the rbNPs rarely changed during storage for at least four weeks (Table 2). These results suggest that rbNPs possess outstanding attributes that meet the criteria for clinical applications.

Various pdNPs exhibit cytotoxic activity against cancer cells. For example, citrus limon-derived NPs suppress mouse tumor growth by activating tumor necrosis factor-related apoptosis-inducing ligand (TRAIL)-mediated apoptosis [45]. Edible tea flower-derived NPs induced high levels of oxidative stress in cancer cells, resulting in mitochondrial damage, cell cycle arrest, and apoptotic cell death [29]. These pdNPs exhibited specific cytotoxicity against cancer cells. In the present study, rbNPs specifically inhibited cancer cell proliferation (Fig. 2A-C). Specifically, rbNPs exhibited the strongest anti-proliferative effect among pdNPs similar in size to rbNPs (Fig. 3A, Supplementary Table S3), suggesting that rbNPs possess a high therapeutic potential for cancer treatment.

We previously reported that cNPs were taken up by colon 26 cells via a lipid raft-mediated pathway [48, 51]. The phospholipid composition of rbNPs was similar to that of cNPs (Supplementary Table S1). Additionally, rice bran contains triglycerides and glycolipids, [52] similar to corn. These results suggest that rbNPs are taken up by cancer cells via a lipid raft-mediated pathway.

Apoptosis is widely known as programmed cell death characterized by morphological changes, including cell shrinkage, chromatin condensation, and DNA fragmentation [53–55]. Over the past decades, most cancer therapeutics have utilized apoptotic mechanisms to eliminate cancer cells [56]. In our study, rbNPs induced DNA fragmentation and chromatin condensation (Fig. 3E and F), indicating that rbNPs induced apoptosis in colon26 cells. Cell cycle arrest occurs before apoptosis [57–59]. We found that rbNPs induced cell cycle arrest in the G2/M phase (Table 3). Phytochemicals in rice bran have been reported to exhibit excellent anti-tumor activity [60, 61]. γ-Tocotrienol, a major phytochemical found in rice bran, inhibits the proliferation of human gastric adenocarcinoma SGC-7901 cells by arresting the cell cycle at the G0/G1 phase [60]. Additionally, γ-oryzanol has been reported to arrest the cell cycle of prostate cancer PC3, LNCaP (at the G2/M phase), and DU145 cells (at the G0/G1 phase) [61]. Supplementary Table S2 shows that these anti-cancer compounds were concentrated in the rbNPs, which may explain why rbNPs are cytotoxic to cancer cells. Rice bran induces an anti-proliferative effect linked to β-catenin-mediated cell proliferation [62, 63]. β-catenin is a protein related to the Wnt signaling pathway, which is known to contribute to cell proliferation and cancer progression [64]. The activated Wnt signaling pathway leads to the expression of proteins involved in cell survival and proliferation, such as cyclins, c-myc, and sequentia [65]. In our study, rbNPs reduced the expression of β-catenin and cyclin D1 in colon26 cells (Fig. 3D) but did not affect β-catenin expression in HaCaT cells (Supplementary Fig. S1C). Furthermore, γ-tocotrienol has been reported to inhibit pancreatic tumors by reducing cyclin D1 [66]. Preventive inositol hexaphosphate extracted from rice bran inhibited colorectal cancer through the Wnt/β-catenin and COX-2 pathways [67]. Taken together, rbNPs may potentially suppress the proliferation of cancer cells by suppressing β-catenin-related pathways and arresting the cell cycle, leading to apoptotic cell death.

Peritoneal dissemination is one of the most unfavorable metastatic forms of gastrointestinal cancer [68–70]. The prognosis of patients with peritoneal dissemination is extremely poor [71, 72], and the survival rate for five years without therapy is only 2% [73]. The delivery of drugs to the peritoneal cavity after systemic administration is limited, making intraperitoneal chemotherapy a reasonable approach for addressing peritoneal metastasis [74–76]. However, intraperitoneal chemotherapy with paclitaxel combined with standard systemic chemotherapy failed [77]. In the present study, we demonstrated that intraperitoneal administration of rbNPs significantly suppressed peritoneally disseminated tumors without causing a decrease in body weight (Fig. 4B and D). The reason for this anti-cancer activity is thought to have resulted from not only the direct cytotoxic activity of rbNPs against colon26 cells (Fig. 2A) but also the production of TNF-α through macrophage activation (Supplementary Fig. S2). After intraperitoneal injection, the DiR-rbNPs remained in the peritoneum for at least six hours and were distributed to multiple abdominal organs. Liposomal doxorubicin remains in the peritoneal cavity longer than doxorubicin alone when administered intraperitoneally [78]. This is because absorption from the abdominal cavity depends on the molecular weight or size of the compound and its solubility [78–80]. The limited absorption of rbNPs into the systemic circulation could explain the limited systemic adverse effects, including the production of inflammatory cytokines, hepatotoxicity, and nephrotoxicity (Fig. 5).

## Conclusions

In conclusion, rbNPs exhibit cancer cell-specific and strong anti-proliferative effects causing significant suppression of peritoneal dissemination and are anticipated to possess potential clinical cancer therapy applications.

### Electronic supplementary material

Below is the link to the electronic supplementary material.

Supplementary Material 1

## Data Availability

All data are available on request.

## Abbreviations

ALT: Alanine aminotransferase
AST: Aspartate aminotransferase
cNPs: Corn-derived nanoparticles
Colon26/fluc: Firefly luciferase (fluc) stably expressing-colon26 murine colon adenocarcinoma
Cre: Creatinine
DiI 1,1′: Dioctadecyl-3,3,3′,3′-tetramethylindocarbocyanine perchlorate
DiI: rbNPs-DiI-labeled rbNPs
DiO 3,3′: Dioctadecyloxacarbocyanine perchlorate
DiO: rbNPs-DiO-labeled rbNPs
DiR 1,1′: Dioctadecyl-3,3,3′,3′-tetramethylindotricarbocyanine iodide
DiR: rbNPs-DiR-labeled rbNPs
DLS: Dynamic light scattering
DMEM: Dulbecco’s modified Eagle’s medium
EVs: Extracellular vesicles
FBS: Fetal bovine serum
Fluc: Firefly luciferase
HRP: Horse radish peroxidase
IL: Interleukin
IS: Internal standard
LC-MS/MS: Liquid chromatography coupled with tandem mass spectrometry
LPCs: Lysophosphatidylcholines
MFI: Mean fluorescence intensity
NPs: Nanoparticles
NT: No treatment
PBS: Phosphate-buffered saline
PC: Phosphatidylcholine
pdNPs: Plant-derived nanoparticles
PE: Phosphatidylethanolamine
PFA: 4% paraformaldehyde phosphate buffer solution
PS: Phosphatidylserine
PSG: Penicillin-streptomycin-l-glutamine solution
PS-Lip: Phosphatidylserine-containing liposomes
rbNPs: Rice bran-derived nanoparticles
rb-sup: Supernatant of the rbNPs
RPMI: Roswell Park Memorial Institute
SDS: Sodium dodecyl sulfate
SMs: Sphingomyelins
TBS: Tris-buffered saline
TBST: TBS with Tween-20
TEM: Transmission electron microscopy
TNF: Tumor necrosis factor
Tween 20: Polyoxyethylene (20) sorbitan monolaurate
