# Supplementary material for: Development of rice bran-derived nanoparticles with excellent anti-cancer activity and their application for peritoneal dissemination
Source: J Nanobiotechnology. 2024 Mar 16;22:114. doi: 10.1186/s12951-024-02381-z (PMC10943818; doi:10.1186/s12951-024-02381-z)
Supplement: Supplementary file 1 — Supplementary Material 1 [file 12951_2024_2381_MOESM1_ESM.docx]

**Supplementary Information**

**Development of rice bran-derived nanoparticles with excellent anti-cancer activity and their application for peritoneal dissemination**

Daisuke Sasaki^1, #^, Hinako Suzuki^1, #^, Kosuke Kusamori^1, 2^, Shoko Itakura^1^, Hiroaki Todo^3^, and Makiya Nishikawa^1^*　　　^#^ These two authors contributed equally to this work.

^1^Laboratory of Biopharmaceutics, Faculty of Pharmaceutical Sciences, Tokyo University of Science, 2641 Yamazaki, Noda, Chiba 278-8510, Japan

^2^Laboratory of Cellular Drug Discovery and Development, Faculty of Pharmaceutical Sciences, Tokyo University of Science, 2641 Yamazaki, Noda, Chiba 278-8510, Japan

^3^Faculty of Pharmacy and Pharmaceutical Sciences, Josai University, 1-1 Keyakidai, Sakado, Saitama 350-0295, Japan

***Corresponding author:** Makiya Nishikawa, Ph.D.

Tel.: +81-4-7121-4450

Fax.: +81-4-7121-4450

E-mail address: makiya@rs.tus.ac.jp

**Supplementary Table S1. Peak area ratio of phospholipids in rbNPs**

| Phospholipids | Acyl chain^*^ | Peak area ratio |
| --- | --- | --- |
|  |  | rbNPs |
| LPC | 18:0 | 58 |
|  | 18:1 | 218 |
|  | 18:2 | 45 |
| PC | 14:0-16:0 | 119 |
|  | 16:0-16:0 | 706 |
|  | 14:0-18:1 | 376 |
|  | 16:0-16:1 | 33 |
|  | 14:0-18:2 | 174 |
|  | 16:0-18:0 | 680 |
|  | 16:0-18:1 | 12982 |
|  | 16:0-18:2 | 6783 |
|  | 16:1-18:1 | 188 |
|  | 16:0-18:3 | 168 |
|  | 16:1-18:2 | 144 |
|  | 18:0-18:0 | 14 |
|  | 18:0-18:1 | 497 |
|  | 18:0-18:2 | 411 |
|  | 18:1-18:1 | 34512 |
|  | 18:1-18:2 | 19679 |
|  | 18:1-18:3 | 299 |
|  | 18:2-18:2 | 8332 |
|  | 18:2-18:3 | 211 |
|  | 18:1-20:0 | 129 |
|  | 18:1-20:1 | 123 |
|  | 18:2-20:0 | 54 |
|  | 18:2-20:1 | 147 |
| LPE | 16:1-18:2 | ND |
| PE | 16:0-16:1 | 13 |
|  | 16:0-18:0 | 108 |
|  | 16:0-20:1 | 2434 |
|  | 18:1-18:1 | 741 |
|  | 18:1-18:2 | 577 |
|  | 18:0-18:1 | 116 |
|  | 18:0-18:2 | 137 |
|  | 18:1-18:1 | 5965 |
|  | 18:1-18:2 | 7637 |
|  | 18:2-18:2 | 3077 |
|  | 18:1-20:0 | 133 |
|  | 18:2-20:0 | 96 |
|  | 18:2-22:0 | 69 |
| PS | 18:0-18:2 | 60 |
|  | 18:1-18:1 | 163 |
|  | 18:1-18:2 | 213 |
|  | 18:2-20:0 | 165 |
| SM | d18:1-20:0 | 154 |
|  | d18:1-22:0 | 205 |
|  | d18:1-22:1 | 228 |

Results are expressed as the mean ± standard deviation (SD); LPC, lysophosphatidylcholine; PC, phosphatidylcholine; LPE, lysophosphatidylethanolamine; PE, phosphatidylethanolamine; PS, phosphatidylserine; SM, sphingomyelin.

*The numbers indicate the carbon number and the degree of unsaturation of the phospholipid acyl chain.

**Supplementary Table S2. Anti-cancer compounds in rb-juice and rbNPs.**

| Anti-cancer compound  (μg/μg protein) | rb-juice | rbNPs |
| --- | --- | --- |
| Ferulic acid | 135.5 ± 1.1 | 151.1 ± 6.7* |
| γ-Oryzanol | 6.0 ± 0.3 | 11.5 ± 2.1* |
| α-Tocopherol | 515.3 ± 0.7 | 948.3 ± 1.6* |
| γ-Tocopherol | 1.3 ± 0.4 | 2.0 ± 0.7 |
| γ-Tocotrienol | 522.0 ± 0.4 | 966.2 ± 0.8* |

The anti-cancer compounds contained in the rb-juice and rbNPs were evaluated by UHPLC-MS (ferulic acid, γ-oryzanol, and γ-tocopherol) or GC-MS (α-tocopherol and γ-tocotrienol). Results are expressed as the mean ± SD of three independent experiments. **p* < 0.05 vs. rb-juice. rbNPs, rice bran-derived nanoparticle.

**Supplementary Table S3. Peak particle size of pdNPs and DOXIL^®^.**

| NPs | Peak particle size (nm) |
| --- | --- |
| Grape NPs | 80.6 ± 20.7 |
| Ginger NPs | 119.3 ± 31.3 |
| Lemon NPs | 95.9 ± 31.1 |
| DOXIL^®^ | 76.0 ± 0.5 |

Grape NPs, ginger NPs, lemon NPs, and DOXIL^®^ were diluted with PBS, and the particle size was measured using Zetasizer. Results are expressed as the mean ± SD of three independent experiments.
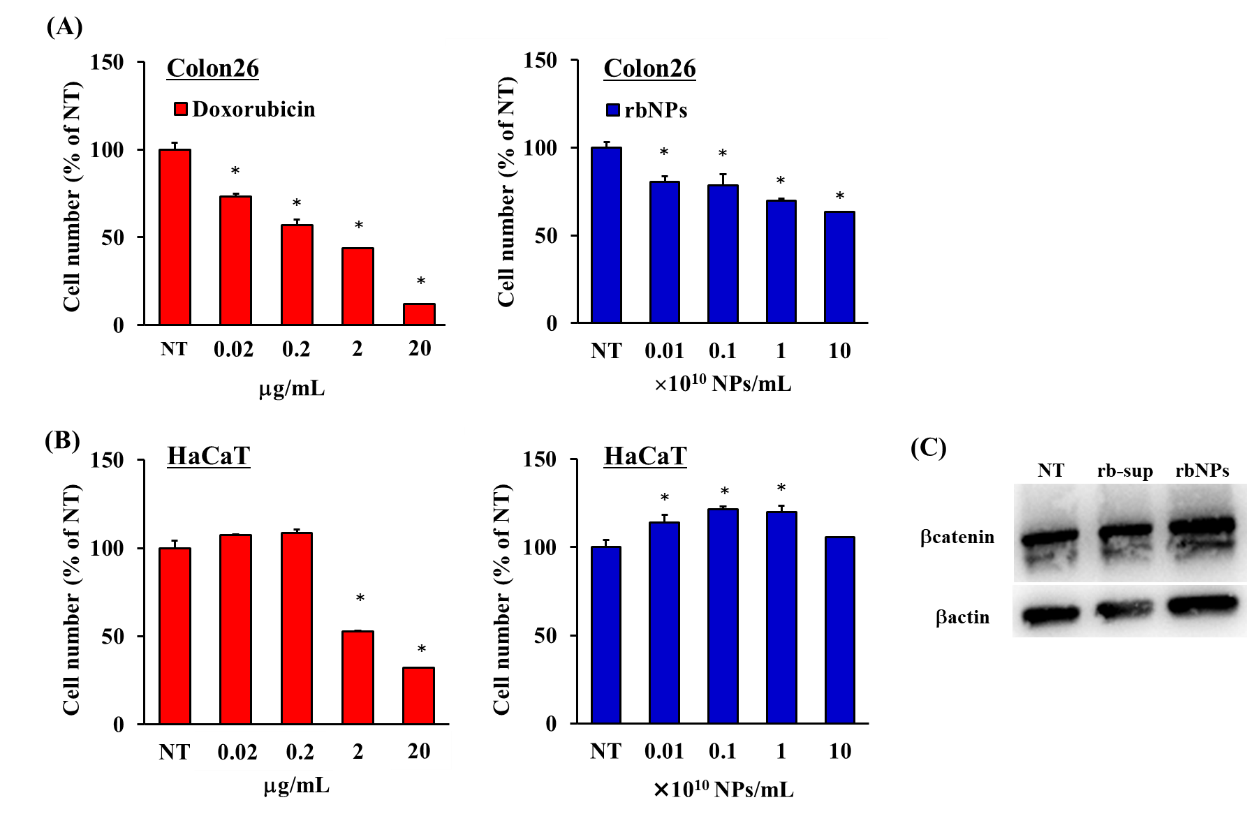


**Supplementary Fig. S1. Cytotoxic activity of rbNPs compared to doxorubicin.**

(A, B) Cell number was measured with CCK-8 assay after 24 h incubation with rbNPs or doxorubicin at different concentrations. Colon26 (A) and HaCaT (B) cells were incubated with 0.01−10×10^9^ rbNPs or 0.02−20 μg/mL doxorubicin. Results are expressed as the mean ± SD of three samples. **p* < 0.05 vs. no treatment (NT) group. CCK-8, cell counting kit-8; rbNPs, rice bran-derived nanoparticle. (C) Western blotting analysis of β-catenin and β-actin in HaCaT cells. HaCaT cells were treated with 1000 μg/mL rbNP or rb-sup for 24 h, and cellular proteins were extracted for analysis. The protein bands were visualized using Invitrogen iBright Imaging Systems. rbNPs, rice bran-derived nanoparticle.

**
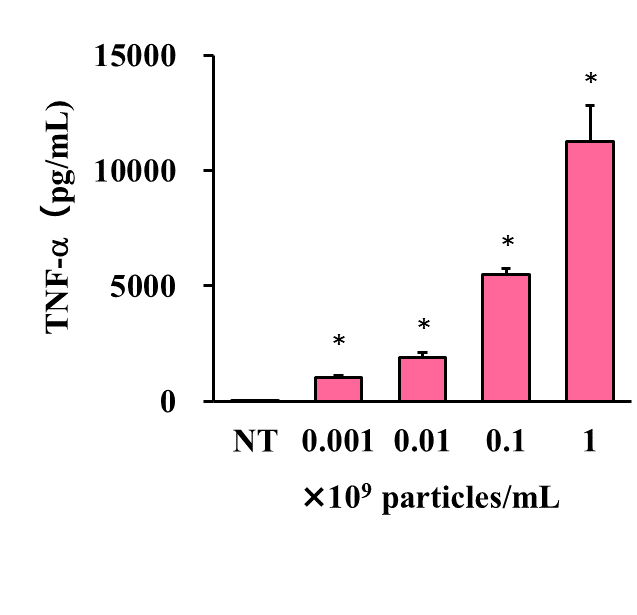
**

**Supplementary Fig. S2. Cytokine release from RAW264.7 cells after adding rbNPs.**

TNF-α release from RAW264.7 cells after adding rbNPs. The supernatants of RAW264.7 cells after 24 h of rbNPs addition were collected for ELISA. Results are expressed as the mean ± SD of three samples. **p* < 0.05 vs. NT. rbNPs, rice bran-derived nanoparticle; TNF-α, tumor necrosis factor-α; ELISA, enzyme-linked immunosorbent assay.
